# Biomarker Potential of Plasma MicroRNA-150-5p in Prostate Cancer

**DOI:** 10.3390/medicina55090564

**Published:** 2019-09-03

**Authors:** Ionut Andrei Paunescu, Razvan Bardan, Anca Marcu, Diana Nitusca, Alis Dema, Serban Negru, Ovidiu Balacescu, Loredana Balacescu, Alin Cumpanas, Ioan Ovidiu Sirbu, Bogdan Petrut, Edward Seclaman, Catalin Marian

**Affiliations:** 1 Department of Urology, Victor Babeș University of Medicine and Pharmacy, Timisoara 300041, Romania; 2 Urology Clinic, Timisoara Emergency County Hospital, Timisoara 300723, Romania; 3 Department of Biochemistry & Pharmacology, Victor Babes University of Medicine and Pharmacy, Timisoara 300041, Romania; 4 Department of Pathology, “Victor Babeş” University of Medicine and Pharmacy, Timisoara 300041, Romania; 5 Department of Oncology, “Victor Babeş” University of Medicine and Pharmacy, Timisoara 300041, Romania; 6 Department of Functional Genomics, Proteomics and Experimental Pathology, The Oncology Institute “Prof. Dr. Ion Chiricuta”, Cluj-Napoca 400015, Romania; 7 Department of Urology, The Oncology Institute “Prof. Dr. Ion Chiricuta”, Cluj-Napoca 400015, Romania

**Keywords:** prostate cancer, microRNA-150-5p, biomarkers, plasma

## Abstract

*Background and Objectives:* Over decades, prostate cancer (PCa) has become one of the leading causes of cancer mortality in men. Extensive evidence exists that microRNAs (miRNAs or miRs) are key players in PCa and a new class of non-invasive cancer biomarkers. *Materials and Methods:* We performed miRNA profiling in plasma and tissues of PCa patients and attempted the validation of candidate individual miRs as biomarkers. *Results:* The comparison of tissue and plasma profiling results revealed five commonly dysregulated miRs, namely, miR-130a-3p, miR-145-5p, miR-148a-3p, miR-150-5p, and miR-365a-3p, of which only three show concordant changes—miR-130a-3p and miR-150-5p were downregulated and miR-148a-3p was upregulated in both tissue and plasma samples, respectively. MiR-150-5p was validated as significantly downregulated in both plasma and tissue cancer samples, with a fold change of −2.697 (*p* < 0.001), and −1.693 (*p* = 0.035), respectively. ROC analysis showed an area under the curve (AUC) of 0.817 (95% CI: 0.680–0.995) for plasma samples and 0.809 (95% CI: 0.616–1.001) for tissue samples. *Conclusions:* We provide data indicating that miR-150-5p plasma variations in PCa patients are associated with concordant changes in prostate cancer tissues; however, given the heterogeneous nature of previous findings of miR-150-5p expression in PCa cells, additional future studies of a larger sample size are warranted in order to confirm the biomarker potential and role of miRNA-150-5p in PCa biology.

## 1. Introduction

Over decades, prostate cancer (PCa) has become one of the leading causes of cancer mortality in men, and the second most common malignancy diagnosed in males worldwide, after skin cancer [1,2]. Furthermore, due to an increase in life expectancy in the general population, the economic burden of prostate cancer is likewise expected to increase. The high mortality rate of PCa is explained by its late diagnosis, because during the early development stages the disease is asymptomatic [2]. Moreover, the diagnostic strategies for this type of malignancy have been proven both inconsistent and non-specific, nurturing the interest in novel, optimized diagnostic and prognostic tools in the field.

Recent studies have provided extensive evidence that microRNAs (miRNAs) are not only key players in PCa, but also a new class of non-invasive cancer biomarkers [3,4]. MiRNAs are small (22–24 nucleotides in length), endogenous non-coding RNA molecules involved in the process of gene silencing through post-transcriptional regulation of target mRNAs and, thus, play a key role in controlling basically all cellular processes associated with cancer biology (proliferation, apoptosis, metastasis, etc.) [5]. Due to their outstanding stability in the body fluids (plasma, serum, urine) of cancer patients, miRNAs have been heavily investigated over the years and proposed as the novel, minimally invasive, optimized biomarkers for the early diagnosis of various malignancies, including prostate cancer [6,7,8].

We investigated herein the relative expression of miRNAs by miRNA profiling in plasma and tissues of PCa patients and attempted the validation of candidate individual miRs as biomarkers for prostate cancer.

## 2. Materials and Methods

### 2.1. Study Design

**Figure 1:**
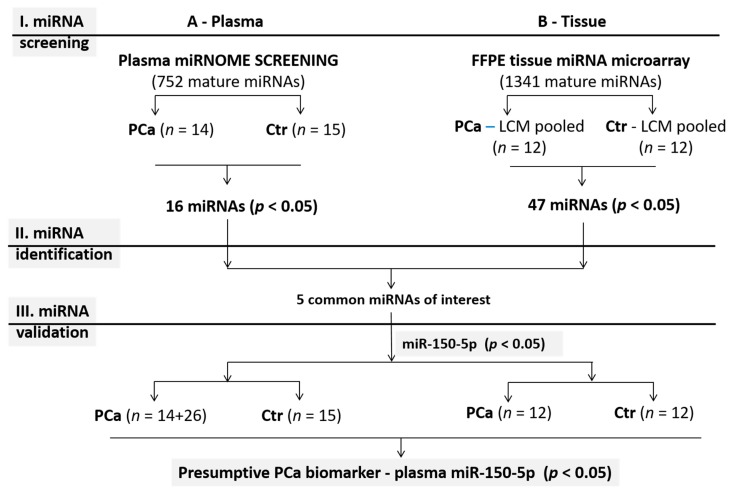
Schematic representation of the study design.

A schematic representation of the study design is presented in Figure 1.

We first performed an miRNA screening in plasma of 14 prostate cancer patients and 15 cancer-free controls, and in the laser capture microdissected (LCM) cancer and adjacent normal tissues of 12 prostate cancer samples. Only five of all differentially expressed miRNAs in both sample types were common, and they were selected for further analysis. Of these, miR-150-5p was validated in the individual plasma samples of 15 controls and 14 plus an additional 26 PCa samples, and the individual 12 cancer and normal tissue samples.

### 2.2. Plasma Samples

Plasma specimens were collected from patients admitted to the Urology Clinic of the Clinical Emergency County Hospital in Timisoara, Romania. All patients had undergone trans-rectal biopsies for histopathological PCa diagnoses. All patients’ clinical data are summarized in Table 1 and extensively presented in Table S1, Supplementary Materials. Cancer-free control samples were collected from subjects with no prostate pathology from the same institution. All controls had normal Prostate-Specific Antigen (PSA) levels (<4 ng/mL) verified by chemiluminescent microparticle immunoassay (Abbott Diagnostics, Lake Forest, IL, USA).

All subjects provided informed consent for use of their biological samples and the study was approved by the Ethics Committees of the participating institutions (the Clinical Emergency County Hospital in Timisoara, code no. 71/05.08.2014 and the Victor Babes University of Medicine and Pharmacy Timisoara, code no. 9/13.05.2014 extended by code no. 33_2017).

Venous blood was collected in EDTA-treated blood collection tubes and was immediately centrifuged for 15 min at 2000× *g* for plasma separation which was subsequently frozen at −80 °C.

### 2.3. Plasma miRNome Screening

We profiled a set of 752 mature miRNAs using the miRCURY™ LNA™ SYBR^®^ Green PCR Kit (Exiqon, QIAGEN, Vedbaek, Denmark) and miRCURY™ LNA™ miRNA miRNome, Human panel I+II, V3.M (Exiqon, QIAGEN, Vedbaek, Denmark). Total RNA was extracted/purified from 200 µL control (*n* = 15) and PCa (*n* = 14) plasma samples using the miRCURY™ RNA Isolation Kit—Biofluids (Exiqon, QIAGEN, Vedbaek, Denmark), according to the manufacturer’s instructions. The same volume (8 µL) from each eluted RNA was reverse transcribed for cDNA synthesis using the miRCURY™ LNA™ miRNA RT Kit (Exiqon, QIAGEN, Vedbaek, Denmark). PCR analysis of multiple miRNAs was simultaneously performed on a 7900 HT Real-Time PCR System (Thermo Fisher Scientific, Waltham, MA, USA).

### 2.4. Plasma miRNA Validation

MiRNA identified as differentially expressed by miRNA screening were further validated using SYBR Green individual assay (miScript Primer Assay/miScript SYBR Green PCR Kit (QIAGEN, Hilden, Germany) on controls (*n* = 15) and PCa samples (*n* = 40, of which 26 were additional samples). RNA was extracted/purified from 200 µl plasma samples using the miRNeasy serum/plasma kit (QIAGEN, Hilden, Germany), according to the manufacturer’s instructions. Samples were spiked with 10 fmoles of cel-miR-39 prior to extraction, to aid in data normalization, as per the manufacturer’s suggestion. cDNA was prepared using the miScript II RT Kit (QIAGEN, Hilden, Germany) and subsequently used as a template in a 7900 HT Real-Time PCR System (Thermo Fisher Scientific, Waltham, MA, USA).

### 2.5. MiRNA Analysis in FFPE Tissues

A set of 12 FFPE prostate adenocarcinoma samples (at least ten-year-old samples, collection of the Department of Pathology, University of Medicine and Pharmacy “Victor Babes” Timisoara) were 10 µm sectioned, mounted on MMI RNAse-free slides (MMI, Zurich, Switzerland) and laser capture microdissected as previously described [9,10]. Total RNA from FFPE laser-microdissected tumor (T) and paired normal (N) tissues was extracted using the miRNAeasy FFPE kit (QIAGEN, Hilden, Germany), according to the manufacturer’s instructions. In this study, 12 pooled tumor RNA samples and 12 pooled normal RNA samples were subjected to microarray analysis in four replicates for each sample type. In all, 100 nanograms of non-fractionated total RNA were directly labeled with Cy3-pCp and hybridized for 20 h at 55 °C on the Unrestricted_Human_miRNA_v16.0 array (Design ID 031181, Agilent Technologies, Santa Clara, CA, USA), using an Agilent miRNA Microarray System with miRNA Complete Labeling and Hyb Kit. Slides were scanned with the Agilent Microarray Scanner G2505C (Santa Clara, CA, USA) and image files were processed with Feature Extraction software v. 11.5.1.1 (Agilent Technologies, Santa Clara, CA, USA).

Individual miRNA validations were performed separately in the same 12 FFPE T and N sample pairs after initial cDNA synthesis (miScript II RT Kit) and miScript Primer Assay as described above.

### 2.6. Statistical Analysis

The data for plasma miRNA profiling were analyzed using the GenEx v.6 (Multid Analyses AB, Gothenburg, Sweden), as suggested by the miRNA screening assay manufacturer (Exiqon). Raw Cq data were preprocessed using as cutoff values 37 for Cq and expression in at least 80% of the samples. Normalizing was done using a global normalization method based on the mean of all expressed miRNAs, as suggested by the assay manufacturer for miRNA screening studies. The relative quantities were log transformed and compared among groups (cancer vs. control) using the Student’s *t*-test.

Individual miRNA data for plasma samples were analyzed by the ΔΔC_t_ method using cel-miR-39 for normalization and RNU6 for FFPE tissue samples, respectively.

Microarray data analysis was assessed in R/Bioconductor (v. 3.5.3, R Foundation for Statistical Computing, Vienna, Austria). Control and flagged spots were systematically removed. Raw median signals were first normalized between arrays with quantile method and then log2 transformed. For each microRNA, a median value of the replicate probes within array was computed. Assessment of microRNAs’ differential expression was done with the limma package (v. 3.38.3, R Foundation for Statistical Computing, Vienna, Austria). Standard errors and log-fold changes between groups were estimated by fitting a linear model to the expression data of each microRNA and empirical Bayes method was used to smooth the standard errors of log-fold changes [11]. Adjusting for the false discovery rate (FDR) was done using the Benjamini–Hochberg procedure.

### 2.7. Bioinformatics Analysis

The predictions of miR targets were performed using the TarPmiR algorithm implemented in miRWalk 3.0 database [12,13]. The miR target list was cross-referenced to the Geo2R GSE21034 data set of differentially expressed miRs (adjusted *p*-value < 0.05) in PCa compared to adjacent normal tissue and then subjected to DAVID gene ontology analysis [14,15].

## 3. Results

The clinical and demographic characteristics of the prostate cancer patients and cancer-free controls are presented in Table 1. No clinical and demographic data are available for the 12 FFPE ten-year-old archived samples; however, they were reviewed by a certified pathologist to confirm the prostate cancer diagnosis.

The plasma screening analysis revealed 16 differentially expressed miRs in cancer versus control samples, of which 10 were downregulated and 6 were upregulated (Table 2).

The FFPE tissue microarray analysis revealed 47 differentially expressed miRs in cancer versus controls, among which 23 were downregulated and 24 were upregulated (Table S2, Supplementary Materials).

The comparison of FFPE tissue and plasma profiling results revealed five commonly dysregulated miRs, namely, miR-130a-3p, miR-145-5p, miR-148a-3p, miR-150-5p, and miR-365a-3p, of which only three show concordant changes—miR-130a-3p and miR-150-5p were downregulated and miR-148a-3p was upregulated in both tissue and plasma samples, respectively. The other two miRs (miR-145-5p and miR-365a-3p) were found to be downregulated in tissue and upregulated in plasma of cancer patients compared to controls (Table 3).

**Figure 2:**
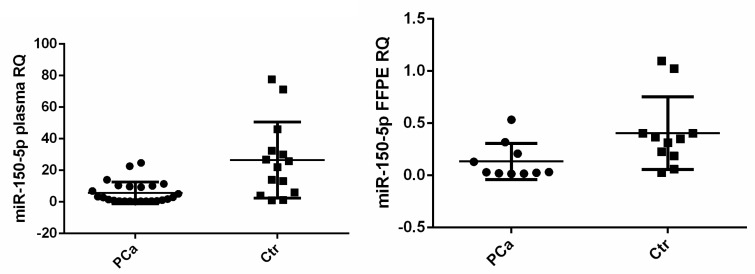
Relative quantities of miR-150-5p in (**left**) plasma and (**right**) FFPE tissue in prostate cancer (PCa) patients (*n* = 40) compared to controls (Ctr) (*n* = 15).

Next, we chose to focus on miR-150-5p individual validation in plasma and tissue samples, considering that its association with PCa is the least known. MiR-150-5p was validated as significantly downregulated in both plasma and FFPE tissue cancer samples, with a fold change of −2.697 (*p* < 0.001) and −1.693 (*p* = 0.035), respectively (Figure 2).

**Figure 3:**
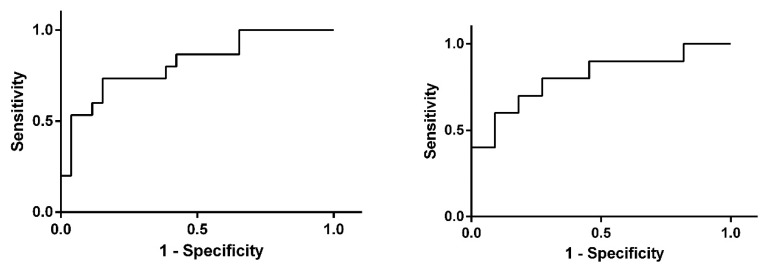
ROC plots of miR-150-5p in (**left**) plasma and (**right**) FFPE tissue in prostate cancer (PCa) patients compared to controls (Ctr).

ROC analysis of miR-150-5p biomarker potential was characterized by an area under the curve (AUC) of 0.817 (95% CI: 0.680–0.995) for plasma samples and 0.809 (95% CI: 0.616–1.001) for FFPE samples (Figure 3).

In order to further understand the biological meaning of miR variations in PCa tissue, we performed DAVID gene ontology analysis on the Geo2R GSE21034 data set of genes (differentially expressed between PCa and adjacent normal tissue) predicted (using the TarPmiR machine learning algorithm) to be targeted by our set of miRs. Altogether, the downregulated miRs impact signaling pathways known for their association with cancer biology in general, and PCa in particular. Of note, miR-150-5p contributes significantly to the modulation of both focal adhesion and extracellular matrix (ECM)–receptor interactions pathways, whereas miR-148 does not pass the 0.05 FDR threshold (Table 4).

## 4. Discussion

We have found herein that miR-148a-3p was upregulated, and that miR-130a-3p and miR-150-5p were significantly downregulated in both tissue and plasma samples from PCa patients compared to cancer-free controls. This confirms recent data showing that miR-148a-3p is upregulated in the tissue and serum of men with PCa, and miR-130a-3p is downregulated in the tissue and urine of PCa patients compared to cancer-free controls [16,17]. However, to the best of our knowledge, there is no data regarding the association of miR-150-5p in biological fluid samples with PCa.

Previous studies regarding miR-150-5p in PCa tissues and cells have revealed inconsistent results. A study by Zhao et al. (2016) reported upregulation of miR-150-5p in PCa cell lines (compared to normal prostate epithelial cells), and functional studies revealed its role in cell proliferation and invasion by modulating p53 expression [18]. Oncogenic miR-150-5p is upregulated in subclones of PCa cells able to resist to chemoradiation therapy or toxic environment, therefore being an indicator of poor prognosis [19].

On the other hand, more recently, it has been suggested that miR-150-5p could function as a potential tumor suppressor in PCa. According to these findings, miR-150-5p inhibits the expression of TRPM4, a gene known to promote the activation of the Wnt/β-catenin signaling (associated with tumorigenesis), and the cell-cycle progression and cell proliferation in several PCa cell lines. Moreover, the upregulation of miR-150-5p and the downregulation of TRPM4 promotes apoptosis and suppresses epithelial–mesenchymal transition, cell migration, invasion, and metastasis by repressing β-catenin signaling [20].

Yet another study suggested that both strands of pre-miR-150 (the guide and passenger strand, miR-150-5p and miR-150-3p, respectively) are downregulated in PCa tissues compared to healthy controls and can act as antitumor miRNAs in naïve and castration-resistant prostate cancer. Of note, transfection of both strands into PCa cell lines leads to a significant inhibition of cell migration and invasion. Moreover, the study proves that SPOCK1 (overexpressed in PCa tissues compared to healthy controls) is a direct target for both strands of pre-miR-150 [21].

Examining 86 prostate cancer tissue samples, Yu et al. (2018) found that miR-150-5p is significantly downregulated in prostate tumor tissue compared to adjacent normal prostate tissue, and that it was associated with poor prognosis in these patients [22]. Furthermore, overexpression of miR-150-5p in PCa cell lines inhibits their proliferation and invasion through regulation of MAP3K12. Of note, focal adhesion, a signaling pathway we predict to be targeted by miR-150-5p in PCa tissue, is also associated with poor prognosis and high aggressiveness in PCa [23]. This is in line with data from other types of human cancers and suggests that miR-150-5p might serve not only as a useful biomarker for PCa progression, but also as a potential therapeutic target [24].

Interestingly, miR-150-5p was reported among the miRNAs downregulated in the prostate tissue of rats with chronic nonbacterial prostatitis, which indicates a possible association with inflammation and/or a possible role in the immune prostate response [25].

To the best of our knowledge, there is no study to investigate the circulating levels of miR-150-5p in PCa patients. Moreover, by performing laser microdissection of the prostate cancer tissues, we reduced the potential of bias induced by the presence of non-cancer cells when measuring miRNA expression in cancer tissues, thus arguing the miRNA-150-5p plasma marker specificity. Nevertheless, the main limitation of our study is the small sample size, and given the heterogeneous nature of previous findings of miR-150-5p expression in PCa cells, additional future studies of a larger sample size are warranted in order to confirm the biomarker potential and role of miRNA-150-5p in PCa biology. In this respect, our results should be viewed within the larger context of biomarker discovery in PCa using multi-omics approaches. It would be interesting to see whether combined with and interpreted in conjunction with PSA/p2PSA/free PSA, sarcosine, or genetic markers (e.g., Prolaris, Oncotype DX), the miR-150-5p could increase the accuracy of (early) diagnostics and its predictive value. Furthermore, there is an obvious need to deepen our understanding of miR-150 association with PCa by comprehensively investigating its impact at the transcriptomic, metabolomic, proteomic, and epigenetic levels, an analytical effort that would need an integrative, complex network approach [26]. Of note, by shedding microvesicles/exosomes, PCa cells have been shown to impact their microenvironment, and to extensively remodel the metabolomic profile in PCa patients; in this respect, it would be interesting to investigate the role of miR-150-5p on PCa lipogenesis, given its reported role as lipid metabolism regulator [27,28,29]. Focused on ex vivo and in vivo PCa models, this multi-omics approach would also be expected to shed light on the role of miR-150-5p as modulator of autophagy, as already outlined in two unrelated cancer models (lung and oral cancers) [30,31]. The importance of this issue is better understood in the context of miR-150-5p’s role as modulator of chemoresistance and overall survival in multiple cancer types (PCa included) [32,33,34].

## 5. Conclusions

We provide data indicating that miR-150-5p plasma variations in PCa patients are associated with concordant changes in prostate cancer tissues. Consequently, we propose miR-150-5p as a possible additional PCa biomarker, which needs to be confirmed by additional validation in larger population samples.

## Figures and Tables

**Table 1:** Clinical and demographic characteristics of the cases and controls.

Characteristics	Cases (*n* = 14)	Controls (*n* = 15)	Cases for Validation (*n* = 26)
Age (years ± SD)	64.9 (±5.45)	51.3 (±8.27)	69.3 (±6.34)
PSA *n* (%)			
<4 ng/mL	0 (0.00)	15 (100.00)	0 (0.00)
4–10 ng/mL	6 (40.00)	0 (0.00)	13 (50.00)
≥10 ng/mL	9 (60.00)	0 (0.00)	12 (46.15)
Gleason Score *n* (%)			
5–6	2 (14.28)		5 (19.23)
7	10 (71.43)		14 (53.84)
**8–10**	**2 (14.28)**		**7 (26.92)**

**Table 2:** Differentially expressed microRNAs between cancer and control plasma samples.

miRs Down	Fold Change	*p*-Value	miRs Up	Fold Change	*p*-Value
miR-130a-3p	−1.4640	0.0006	miR-195-5p	4.4277	0.0011
miR-342-3p	−1.4793	0.0045	miR-148a-3p	1.6332	0.0084
miR-20b-5p	−2.6733	0.0048	miR-145-5p	1.6182	0.0092
miR-150-5p	−1.6961	0.0091	let-7c	1.5653	0.0213
miR-324-3p	−1.5342	0.0108	miR-365a-3p	1.6709	0.0350
miR-376a-3p	−2.6422	0.0160	miR-199b-5p	2.1282	0.0463
miR-93-5p	−1.3391	0.0273			
miR-7-5p	−1.7245	0.0392			
miR-29b-3p	−1.3472	0.0418			
miR-215	−1.4886	0.0433			

**Table 3:** Common differentially expressed miRs among tissue and plasma screening.

miR	FFPE Microarray		Plasma Screening	
	FC	*p*-Value	FC	*p*-Value
miR-148a-3p	1.647	0.012	1.633	0.008
miR-130a-3p	−1.618	<0.001	−1.468	0.0005
miR-150-5p	−1.409	0.002	−1.696	0.009
miR-145-5p	−1.748	0.020	1.618	0.009
miR-365a-3p	−1.412	0.004	1.670	0.035

**Table 4:** KEGG pathways predicted to be targeted by the miRs found dysregulated.

	Term (KEGG Pathway)	*p*-Value	Fold Enrichment	FDR
miR-130a-3p, miR-150-5p, miR-145-5p, miR-365a-5p	hsa04510: Focal adhesion	1.68 × 10^−12^	2.133	2.24 × 10^−9^
	hsa05200: Pathways in cancer	6.35 × 10^−9^	1.636	8.44 × 10^−6^
	hsa04512: ECM–receptor interaction	5.77 × 10^−8^	2.402	7.67 × 10^−5^
	hsa04722: Neurotrophin signaling pathway	5.58 × 10^−6^	1.964	7.42 × 10^−3^
	hsa04070: Phosphatidylinositol signaling	6.28 × 10^−6^	2.077	8.35 × 10^−3^
	hsa04151: PI3K-Akt signaling pathway	2.18 × 10^−5^	1.491	2.89 × 10^−2^
	hsa05222: Small-cell lung cancer	2.81 × 10^−5^	2.079	3.73 × 10^−2^
miR-130a-3p, miR-150-5p	hsa05200: Pathways in cancer	1.30 × 10^−6^	1.795	1.72 × 10^−3^
	hsa04510: Focal adhesion	2.43 × 10^−6^	2.134	3.21 × 10^−3^
	hsa04070: Phosphatidylinositol signaling	1.60 × 10^−5^	2.608	2.12 × 10^−2^
miR-150-5p	hsa04510: Focal adhesion	2.27 × 10^−9^	2.322	3.00 × 10^−6^
	hsa04512: ECM–receptor interaction	2.58 × 10^−6^	2.699	3.42 × 10^−3^
miR-148a-3p	hsa04512: ECM–receptor interaction	5.08 × 10^−3^	1.379	6.40 × 10^−1^
	hsa04510: Focal adhesion	2.26 × 10^−2^	2.069	2.57 × 10^1^

Note: FDR, false discovery rate adjusted *p*-value.

## Supplementary Materials

The following are available online at https://www.mdpi.com/1010-660X/55/9/564/s1, Table S1: Clinical characteristics of the individual patients, Table S2: Differeantially expressed microRNAs in cancer versus normal tissue.
